# DIA-MS Based Proteomics Combined with RNA-Seq Data to Unveil the Mitochondrial Dysfunction in Human Glioblastoma

**DOI:** 10.3390/molecules28041595

**Published:** 2023-02-07

**Authors:** Hao-Long Zeng, Lizhi Hu, Xi Chen, Qiang-Qiang Han, Huijun Li, Liming Cheng, Chao-Xi Li

**Affiliations:** 1 Department of Laboratory Medicine, Tongji Hospital, Tongji Medical College, Huazhong University of Science and Technology, Wuhan 430030, China; 2 Clinical Center for Human Genomic Research, Union Hospital, Huazhong University of Science and Technology, Wuhan 430030, China; 3 SpecAlly Life Technology Co., Ltd., Wuhan 430030, China; 4 Department of Neurosurgery, Tongji Hospital, Tongji Medical College, Huazhong University of Science and Technology, Wuhan 430030, China

**Keywords:** glioblastoma, mitochondria, proteomics, transcriptomics, OXPHOS

## Abstract

Mitochondrial dysfunctions underlie the pathogenesis in glioblastoma multiforme (GBM). Comprehensive proteomic profiling of mitochondria-specific changes in human GBM is still insufficient. This study carried out a DIA-MS based proteomic analysis on the mitochondria isolated from human primary GBM and peritumoral tissue (as paired control), and further compared those findings with the transcriptomic datasets. A total of 538 mitochondrion-specific proteins were rigorously confirmed, among which 190 differentially expressed proteins were identified. Co-regulations of the mitochondrial dysfunction pathway networks were observed, including significant up-regulations of mitochondrial translation and apoptosis, as well as down-regulations of OXPHOS and mitochondrial dynamics. Proteins related to FA, AA metabolism and ROS also showed significant variations. Most of these alterations were consistent in trend when compared the proteomics findings with the RNA-Seq datasets, while the changes at protein levels appeared to be more dramatic. Potentially key proteins in GBM were identified, including up-regulated pro-apoptotic protein CASP3, BAX, fatty acid oxidation enzymes CPT1A, CPT2, ACADM, serine-glycine enzymes SHMT2, GATM, ROS-related protein SOD2, GPX1, and CAT; and down-regulated dynamin-related protein MFN1, MFN2, OPA1, and OXPHOS components; and also several differentially expressed ALDH isoforms. This study systematically profiled the mitochondrial dysfunctions by combining proteomic findings and mRNA datasets, which would be a valuable resource to the community for further thorough analyses.

## 1. Introduction

Glioblastoma multiforme (GBM) is the most common primary malignant brain tumor, with median survival under 2 years [1]. Although several treatment options are available, including surgery, along with adjuvant chemo- and radio-therapy, the disease has a poor prognosis [2]. New molecular biomarkers are urgently needed to improve diagnosis, prognostication, and therapeutic options [3]. Nevertheless, mechanisms associated with the pathogenesis involves aberrations of multiple signaling pathways through genetic mutations and altered gene expression have yet to be completely understood.

Mitochondria are a central hub of energy metabolism and play critical roles in redox balance, oncogenic signaling, innate immunity, and apoptosis. Mitochondria also undergo constant morphological changes through fusion and fission, which is a highly regulated process referred as mitochondrial dynamics [4]. A shred of evidence indicates that mitochondrial dysfunction supports tumor initiation and advancements, promotes chemoresistance, inhibits apoptosis [5], among which, the Warburg effect is one of the most studied abnormalities. In GBM, genetic events that drive aberrant cancer cell proliferation alter mitochondrial metabolism, including promoting aerobic glycolysis, but do not typically impair mitochondrial function [6]. Previous studies have demonstrated some aspects of the mitochondrial metabolic alterations including oxidative phosphorylation (OXHPOS), and mitochondrial dynamics [7]; however, systematic profiling of the mitochondria-specific changes involving multiple signaling pathways are still insufficient.

In this study, we isolated mitochondria from GBM and its paired peritumoral tissue in patients with histopathologically confirmed GBM, and subjected for DIA-MS proteomics analysis, and then combined the data with the transcriptional changes to profile the mitochondrial dysfunctions at both protein and mRNA level. This study would provide a useful resource to the community for further thorough analyses, including elucidating the pathological mechanism, or developing new therapeutic interventions or drugs for GBM.

## 2. Results

### 2.1. Mitochondrial Proteome Alterations

**Figure 1:**
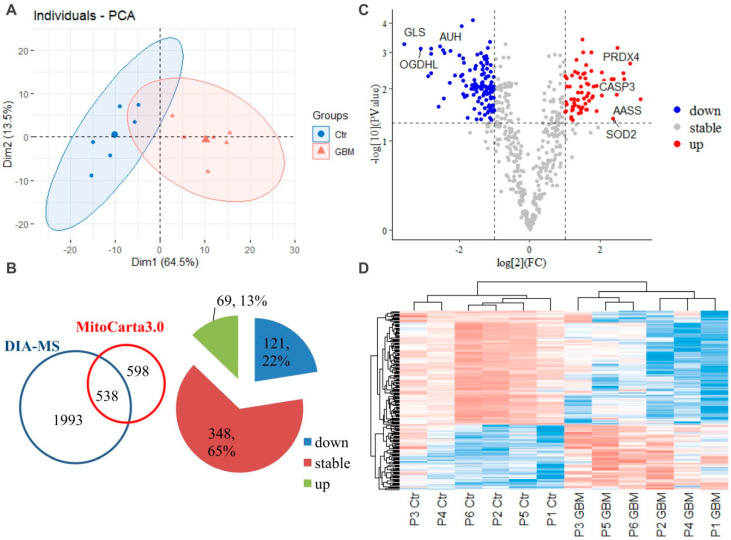
DIA−MS based proteomics identified mitochondria-specific changes in GBM. (**A**) PCA of the individual samples from the GBM and control (Ctr) group for all the 2531 successfully quantified proteins (Table S2). (**B**) a total of 538 mitochondrion-specific proteins were confirmed by mapping with the MitoCarta3.0 (Table S3), among which 190 differentially expressed proteins were identified. (**C**) volcano plot of differentia mitochondrion-specific proteins according to the cutoff threshold of log_2_(FC) >1 or <−1 and *p* < 0.05. (**D**) A hierarchical heatmap between the GBM and control (Ctr) samples for all the patients (P1-P6). Abbreviations: CASP3, Caspase-3; SOD2, Superoxide dismutase; PRDX4, Peroxiredoxin-4; AASS, Alpha-aminoadipic semialdehyde synthase; OGDHL, 2-oxoglutarate dehydrogenase; GLS, Glutaminase; AUH, Methylglutaconyl-CoA hydratase.

To identify the mitochondrial dysfunction, we firstly employed a DIA-MS based quantitative proteomics strategy to analyze the alterations of the isolated mitochondrion from GBM tumor and the paired peritumoral tissue (as control) samples from six patients diagnosed with GBM. After a strict data filtering, we totally quantified 2531 proteins in all GBM and control samples, which has been listed in Table S2. PCA showed a clear discrimination between the individuals of the GBM and control group (Figure 1A). In order to extract the mitochondrion-specific proteins, MitoCarta3.0, a catalogue of gene symbols for human mitochondrion, was used for filtration. A total of 538 mitochondria-specific proteins were confirmed (Figure 1B, Table S3), among which 190 differentially expressed proteins (DEP) were identified, according to the cutoff threshold of log_2_(FC) >1 or <−1 and *p* < 0.05 (Figure 1B,C). Of these DEPs, 69 (13%) and 121 (22%) were identified as up- and down-regulated proteins, respectively. Some potentially key functional proteins were identified here, as labeled in the volcano plot (Figure 1C), such as up-regulated protein CASP3 (Caspase-3), SOD2 (Superoxide dismutase), PRDX4 (Peroxiredoxin-4), AASS (Alpha-aminoadipic semialdehyde synthase), and down-regulated protein OGDHL (2-oxoglutarate dehydrogenase), GLS (Glutaminase), and AUH (Methylglutaconyl-CoA hydratase). The further hierarchical heatmap showed that the DEPs had markedly different proteomic patterns between the GBM and control samples (Figure 1D).

**Figure 2:**
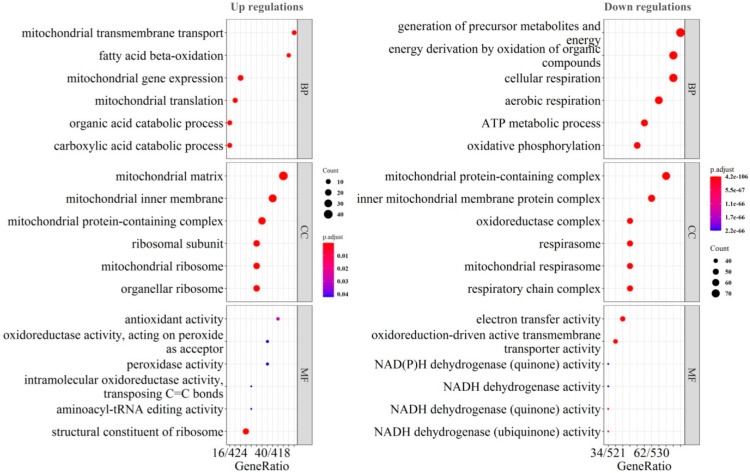
Gene Ontology (GO) enrichment analysis of the differentia proteins. Biological process (BP), cellular component (CC), and molecular function (MF) categories were listed separately for the up- and down-regulated proteins, respectively.

To further characterize the functional identity of the DEPs, gene ontology (GO) enrichment analysis was performed (Figure 2). Biological process (BP), cellular component (CC), and molecular function (MF) categories were listed separately for the up- and down-regulated proteins, respectively. In the aspect of BP, mitochondrial translation, fatty acid beta-oxidation, and organic acid catabolism were up-regulated, while energy metabolisms including cellular respiration, ATP metabolism and oxidative phosphorylation (OXPHOS) were down-regulated. In the aspect of CC, mitochondrial ribosome was up-regulated, while mitochondrial respirasome was down-regulated. In the aspect of MF, antioxidant activity, peroxidase activity, structure constitute of ribosome were up-regulated while NADP dehydrogenase activity was down-regulated. All the BP, CC and MF results suggested the consistent and significant up-regulations in mitochondrial translation and down-regulation in OXPHOS. In addition, the up-regulation in fatty acid beta-oxidation or peroxide metabolism also could be observed.

**Figure 3:**
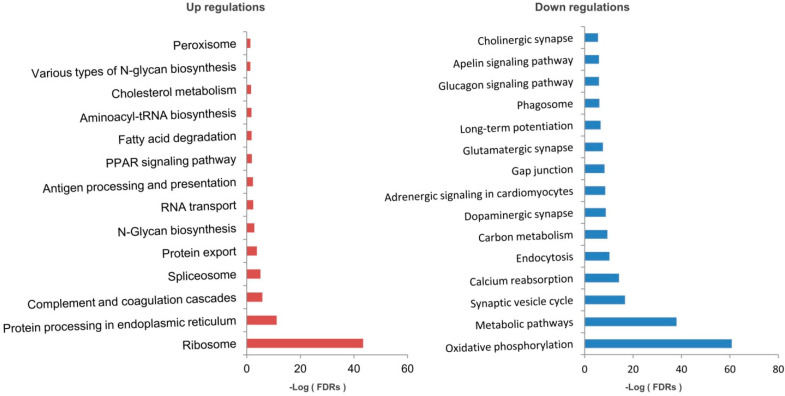
KEGG pathway enrichment analysis of the differentia proteins. The top up−regulations and down−regulations enriched pathways were listed separately. FDRs, false discovery rates.

The DEPs were further subjected to KEGG pathway enrichment analysis (Figure 3). The top up-regulated pathway include ‘Ribosome’, ‘Protein processing in endoplasmic reticulum’, ‘Complement and coagulation cascades’ and ‘Spliceosome’, while top down-regulated pathway include ‘Oxidative phosphorylation (OXPHOS)’, ‘Metabolic pathways’, ‘Synaptic vesicle cycle’ and ‘calcium reabsorption’. These findings were mostly consistent with the above GO enrichment findings.

### 2.2. Transcriptomic Alterations of the Mitochondria-Specific Proteins

**Figure 4:**
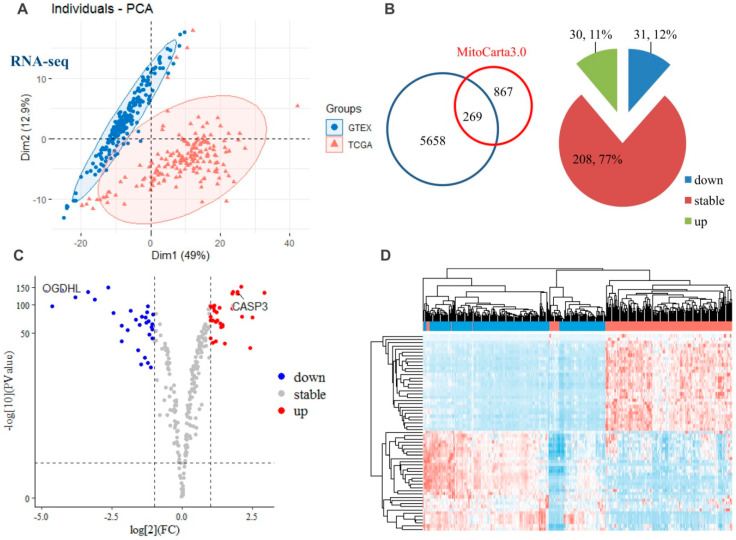
Transcriptional alterations of the mitochondrial-specific proteins. (**A**) PCA on the total gene expressions of 166 samples of TCGA GBM tumor and 206 samples of GTEx normal brain tissues. (**B**) A total of 269 mitochondrion-specific proteins were confirmed by mapping with the MitoCarta3.0, among which 30 (11%) and 31 (12%) genes were identified as up- and down-regulated genes. (**C**) Volcano plot of differentia mitochondrion-specific proteins according to the cutoff threshold of log_2_(FC) >1 or <−1 and *p* < 0.05. (**D**) A hierarchical heatmap between the GBM samples (red labels at top) and normal samples (blue labels at top). Abbreviations: CASP3, Caspase-3; OGDHL, 2-oxoglutarate dehydrogenase.

By using the publicly available TCGA and GTEx RNA-Seq database as the primary sources of GBM and normal brain tissue transcriptomic analysis, we obtained a total of 5927 gene expressions on 166 samples of GBM tumor and 206 samples of normal brain tissues. PCA showed a clear discrimination between the individuals of the GBM and control group (Figure 4A). After filtering by using MitoCarta3.0, a gene catalogue of human mitochondrion, 269 mitochondrial proteins retained (Figure 4B), among which 30 (11%) and 31 (12%) genes were identified as up- and down-regulated genes, respectively, in GBM when compared to normal brain tissue. (Figure 4B,C). CASP3 (Caspase-3), and OGDHL (2-oxoglutarate dehydrogenase) were found to be up- and down-regulated, respectively. The hierarchical heatmap showed that the differentially expressed mitochondrial genes had markedly different patterns between the GBM and normal (GTEx) groups (Figure 4D).

### 2.3. Mitochondrial Dysfunction by Integrated Proteomic and RNA-Seq Findings

**Figure 5:**
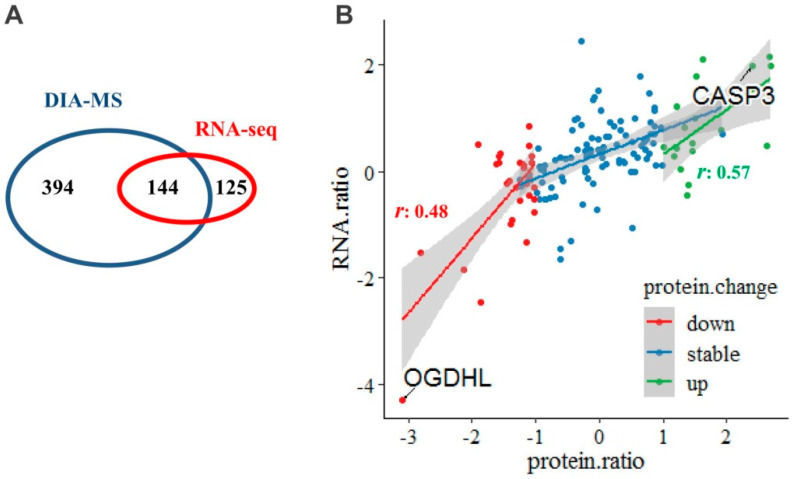
Correlations of the changed mitochondria-specific proteins between protein and mRNA level. (**A**) A total of 144 mitochondrial proteins were overlapped when integrated the proteomic and RNA-seq data. (**B**) A separate scatter diagram was plot for the up- and down-regulated proteins/genes. The Pearson correlation coefficients (*r*) was 0.57 and 0.48, respectively (*p* < 0.05).

When integrated the proteomic and RNA-seq data, 144 mitochondria-specific proteins were overlapped (Figure 5A, Table S4). A separate scatter diagram was plot and showed that, the up- and down-regulated proteins/genes were significantly positive-correlated, respectively, between protein and RNA levels, with *r* (Pearson correlation coefficient) of 0.57 and 0.48 (Figure 5B).

**Figure 6:**
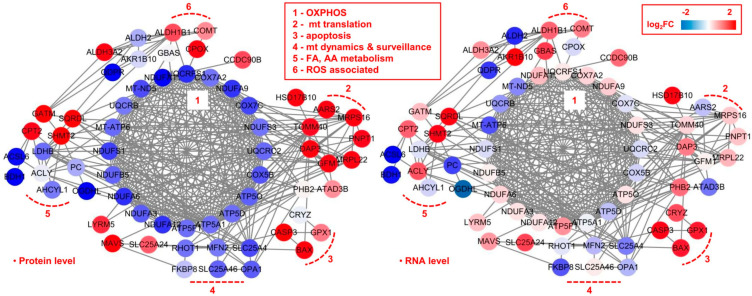
Interaction network based on mitochondria-specific proteins differentially expressed at protein or mRNA level in GBM. Proteins that differentially expressed either at protein level or mRNA level (N = 65) were extracted and subjected for network analysis. The fold-change based protein–protein interaction (PPI) network at protein level and at RNA level, were established, respectively, which mainly consisted of six functional clusters: (1) oxidative phosphorylation (OXPHOS), (2) mitochondrial (mt) translation, (3) apoptosis, (4) mt dynamics and surveillance, (5) fatty acid (FA), amino acid (AA) metabolism, (6) reactive oxygen species (ROS) associated. Node color represents the quantitative ratio.

We extracted all the proteins that differentially expressed either at protein level or at mRNA level (N = 65), and subjected them for network analysis. Based on the functional annotation in MitoCarta3.0, we established the fold-change based protein–protein interaction (PPI) network, at protein level and at mRNA level, respectively (Figure 6). We found that the PPI network consisted of proteins/genes in mainly six functional clusters: (1) oxidative phosphorylation (OXPHOS), (2) mitochondrial (mt) translation, (3) apoptosis, (4) mt dynamics and surveillance, (5) fatty acid (FA), amino acid (AA) metabolism, (6) reactive oxygen species (ROS) associated.

As a whole, when compared the PPI network at protein level to that at mRNA level, we found that most of the changes in proteins production and genes expression are consistent with each other. Specifically, at the protein level, OXPHOS and mitochondrial dynamics and surveillance showed a markedly downward co-regulation, while mitochondrial translation and apoptosis showed markedly upward co-regulations. While for the FA and AA metabolism, and ROS associated functional clusters, both up- and down-regulated proteins could be observed. More specifically, in the FA and AA metabolism functional cluster, SHMT2, CPT2, GATM and SQRDL were up-regulated, while other protein such as OGDHL, ACSL6, BHD1 were down-regulated. In the ROS associated functional cluster, CPOX, ALDH3A2 were up-regulated while QDPR was down-regulated.

At mRNA level, OXPHOS and mitochondrial translation seems not to change as much as that at protein level. While apoptosis was significantly up-regulated and mitochondrial dynamics and surveillance was significantly down-regulated, which was in good consistency with the protein level.

### 2.4. Possible Signatures in Human GBM Mitochondrion

**Figure 7:**
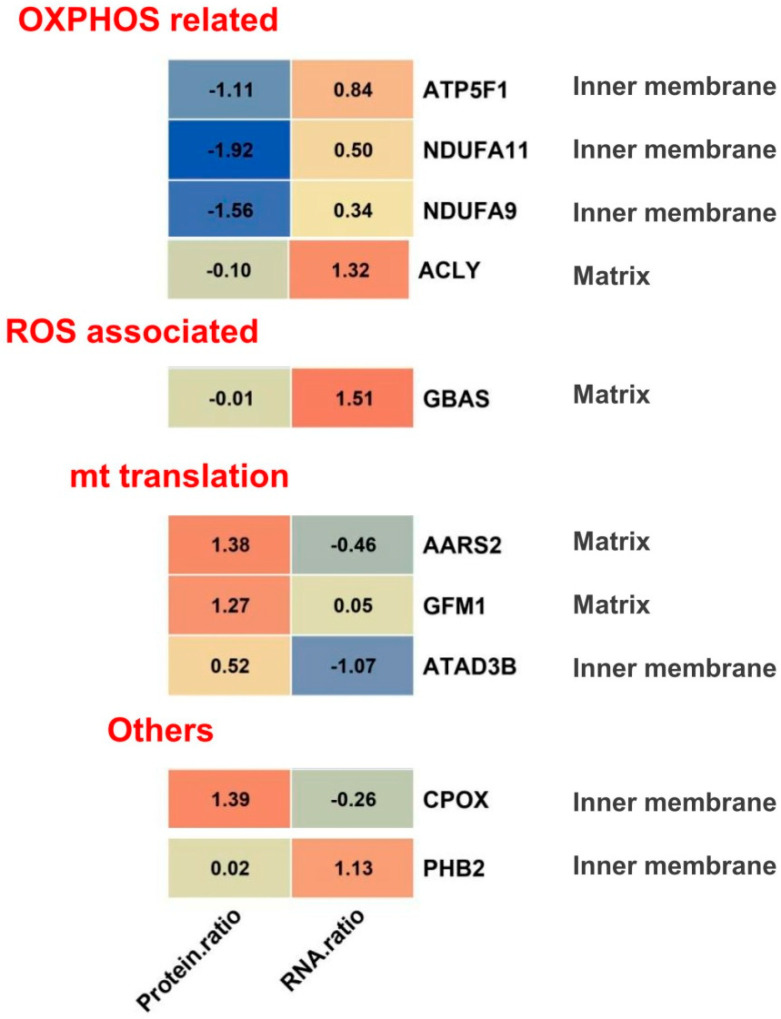
Colormap of proteins that changed inconsistently at the protein and mRNA levels. A total of 10 proteins were selected, including the OXPHOS related proteins ATP5F1, NDUFA11, NDUFA9, ACLY, and ROS associated protein GBAS, and mitochondrial translation related protein AARS2, GFM1, ATAD3B, and other protein CPOX, PHB2. Their sub-mitochondrion localization were mitochondrial inner membrane or matrix.

In a cell, the relationship between protein and mRNA are mainly determined by translation and protein degradation—two processes that are highly regulated both at a global and at a gene-specific level. Their translation differences or deregulation can lead to diverse diseases, including cancer, such as GBM [8]. Therefore, we focused on proteins that had markedly discordant changes between the protein level and RNA level. A total of 10 proteins were selected and listed in Figure 7, including the OXPHOS related proteins ATP5F1, NDUFA11, NDUFA9, ACLY, and ROS associated protein GBAS, and mitochondrial translation related protein AARS2, GFM1, ATAD3B, and other protein CPOX, PHB2. Their sub-mitochondrion localization were mitochondrial inner membrane or matrix. These changed proteins with discordant variations between protein and m RNA levels may have special functions in GBM, which still need to be deeply clarified in future.

**Figure 8:**
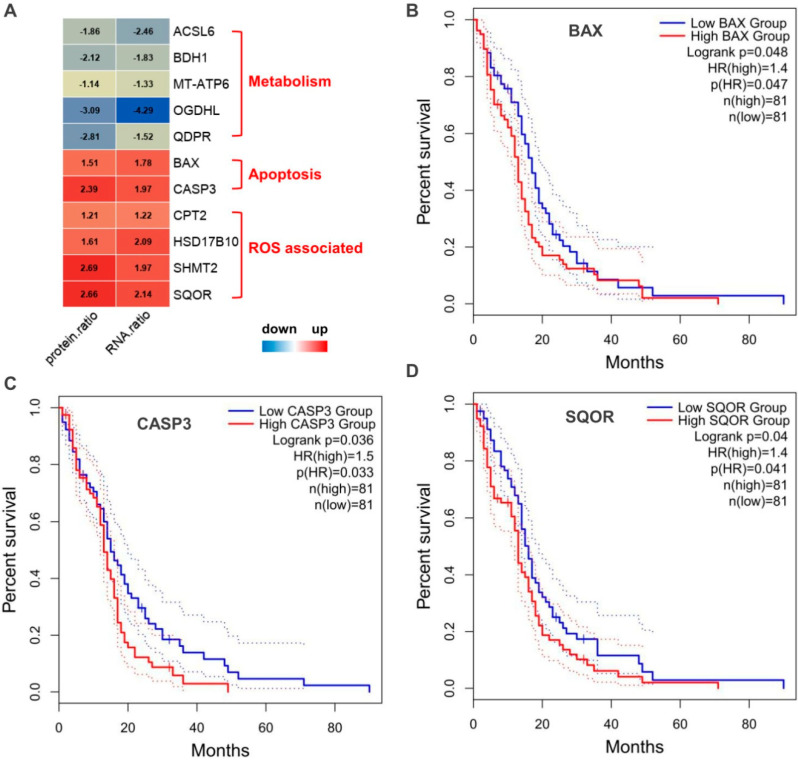
Potential molecular signatures in human GBM. (**A**) Colormap of the proteins that differentially expressed both at protein and mRNA levels. (**B**–**D**) Kaplan–Meier survival analysis of patients between the group of high- and low- expression level of CASP3, BAX, or SQOR in the TCGA. Abbreviations: HR, Hazard Ratio; CASP3, Caspase-3; BAX, Apoptosis regulator BAX; SQOR, Sulfide: quinone oxidoreductase.

In order to find the possible molecular signatures in human GBM, we also focused on proteins that differentially expressed both at protein and mRNA levels. A total of 11 proteins were identified (Figure 8A), which were all changed consistently between protein and mRNA level. The metabolism related protein ACSL6, BDH1, MT-ATP6, OGDHL, and QDPR were down-regulated, while apoptosis related protein BAX, CASP3, and ROS associated protein CPT2, HSD17B10, SHMT2, and SQRDL were up-regulated. We subjected these proteins to the TCGA-GBM transcriptomic database for survival analysis, only CASP3, BAX, and SQOR showed significances (*p* < 0.05) (Figure 8B–D). Patients in the group of low expression level of CASP3, BAX, or SQOR, were all accompanied by higher OS (Overall Survival) than patients in the group of high expressions. The HR (Hazard Ratio) value were 1.5, 1.4, 1.4 for CASP3, BAX, and SQOR, respectively.

## 3. Discussion

Mitochondria play a central and multifunctional role in malignant tumor progression, thus targeting mitochondria provides therapeutic opportunities [6]. It has been known that genetic events that drive aberrant cancer cell proliferation also alter the mitochondrial bioenergetic and biosynthetic state, including promoting aerobic glycolysis, but not typically impair mitochondrial function [9]. In the present study, we isolated the mitochondria from GBM and peritumoral tissue in patients with histopathologically confirmed GBM, and subjected for DIA-MS based proteomics analysis. As expected, the mitochondrial proteome showed different expression patterns between GBM and peritumoral controls, suggesting significant mitochondrial dysfunctions in GBM, which involved up-regulations of mitochondrial translation and apoptosis, as well as down-regulations of OXPHOS and mitochondrial dynamics and surveillance. In addition, proteins related to FA, AA metabolism and ROS also showed significant variations in GBM. Furthermore, when we compared these proteomic findings with the mRNA level data, most of the alterations were consistent except for a few proteins/genes, and the changes in protein levels seems to be relatively more dramatic. This implied that mitochondrial alterations may mostly occurred in transcriptional levels but possibly amplified in translational level. In mitochondria, the altered biological state communicate with the nucleus through mitochondrial ‘retrograde signaling’ to modulate signal transduction pathways, transcriptional circuits, and chromatin structure to meet the perceived mitochondrial and nuclear requirements of the cancer cell [9], which may benefit for the formation of tumor microenvironment.

### 3.1. OXPHOS

Hypoxia is known to drive the aggressive character of glioblastoma multiforme by promoting aerobic glycolysis rather than pyruvate oxidation in OXPHOS, a phenomenon termed the Warburg effect, which is a general feature of oncogenesis [7]. As expected, our data revealed that OXPHOS proteins showed significant downward co-regulations in GBM, suggesting OXPHOS capacity is markedly reduced. However, compared to the down-regulations at the protein level, changes at mRNA levels seemed to be much less, and some genes even showed discordant changes, such as ATP5F1, NDUFA11 et al. Because the mitochondrial OXPHOS in GBM is intact in function, and OXPHOS still plays an essential role in tumorigenesis and tumor progression, we supposed that the dramatic proteomic alterations may result from combined various factors, such as oncogenes, tumor suppressors, and a hypoxic microenvironment [10].

### 3.2. Mitochondrial Dynamics

Mitochondrial dynamics tightly correlate with OXPHOS activity and mitochondrial membrane potential: fusion requires high potential and active OXPHOS, while fission is induced by uneven mitochondrial membrane potential and damaged OXPHOS [7]. In consistent with the changes in OXPHOS, our data revealed the proteins related to mitochondrial dynamics showed significant downward co-regulations in GBM, especially for the three dynamin-related proteins regulate mitochondrial fusion: MFN1, MFN2, and OPA1. As mitochondrial fusion suppresses tumor progression [5], we supposed the down-regulation of mitochondrial fusion proteins in GBM may contribute to the tumorigenesis or progression.

### 3.3. Mitochondrial Translation

Mitochondrial translation is essential for the function of glioblastoma stem cells (GSCs), which rely highly on OXPHOS [11]. The mitochondrial translation machinery is up-regulated in a subset of human tumors [12]. In consistent with these reports, our data revealed that proteins related to the mitochondrial translation were significantly upward co-regulated, and these proteins were significantly enriched in the ribosome related biological processes and pathways. Because proteins encoded in the mitochondrial DNA (mtDNA), which are all OXPHOS components, are synthesized in specialized mitochondrial ribosomes (mitoribosomes), the mitoribosomes are therefore essential for cellular respiration and tumorigenesis or tumor progression [12]. Inhibition of mitochondrial translation suppresses glioblastoma stem cell growth [11], suggesting mitochondrial translation could be explored as a therapeutic target. In addition, compared to the up-regulations at protein level, the changes in mitochondrial translation at mRNA level seemed to be much less or not significant, such as protein AARS2, GFM1, suggesting the alterations may mostly occurred or amplified in translational process, which still needs to be clarified in future studies.

### 3.4. Apoptosis

The increasing body of literature has reported an alternative role of several mitochondrial ribosomal proteins as apoptosis-inducing factors [11]. Apoptotic death in the core of glioblastoma multiforme is triggered by hypoxia and lack of nutrients, with the over-expression of pro-apoptotic BAX (apoptosis regulator), upregulation of CASP3 (caspase-3), and occurrence of internucleosomal DNA fragmentation [13]. As expected, our data revealed that the apoptosis related proteins, including CASP3, BAX, were significantly increased in GBM at both protein and mRNA level, and high CASP3 and BAX expression was associated with shorter survival in GBM. Higher levels of activated CASP3 in tumor tissues are correlated with significantly increased rate of recurrence and deaths, as activated CASP3 potently stimulates the repopulation of surviving tumor cells [14].

### 3.5. ROS

Reactive oxygen species (ROS) are important byproducts of cell metabolism. The maintenance of ROS homeostasis is crucial for cancer cell survival. The most important ROS detoxification mechanisms include superoxide dismutase (SOD2), catalase (CAT), and glutathione peroxidase (GPX) [15]. Our proteomic data showed that SOD2, GPX1, CAT all up-regulated in GBM, suggested up-regulation of ROS detoxification occurred.

In addition, cancer cells preferentially utilize cytosolic NADH supplied by aldehyde dehydrogenase (ALDH) for ATP production through OXPHOS [16]. Our data identified several ALDH isoforms to be differentially expressed in GBM, including up-regulated ALDH1B1, ALDH3A2, and down-regulated ALDH2, ALDH5A1. ALDH isoforms are reported to be highly up-regulated in non-small cell lung cancer, and several ALDH isoforms are also increased in GBM, leading us to hypothesize that GBM metabolism is dependent on ALDH [16]. ALDH1 is overexpressed in stem-like cancer cells in GBM, and aberrant high ALDH1 activity correlated with poor prognosis and more aggressive cancer biology [17].

### 3.6. FA and AA Metabolism

GBM acquires large amounts of free fatty acids (FAs) to promote cell growth [18]. Our data revealed that mitochondrial fatty acid oxidation (FAO) enzymes CPT1A, CPT2, and ACADM were up-regulated in GBM significantly. These three proteins has been reported to be increased in recurrent GBM patients with poor prognosis, and the enhanced fat acid metabolism promotes aggressive growth of GBM with CD47-mediated immune evasion [19]. Except that, we also found ACSL6, a member of the long-chain acyl-CoA synthetase family, was found to be decreased in GBM, which has been observed in many forms of cancers, except in CRC [20], but the mechanisms still need to be clarified.

For amino acid (AA) metabolism, we found SHMT2 (serine hydroxymethyltransferase 2) were significantly increased in GBM. SHMT2 catalyzes the conversion of serine to glycine and one-carbon transfer reactions in mitochondria, plays a key role in the survival of brain cancer cells within the ischemic zones of gliomas [21]. We also found that glycine amidinotransferase (GATM), the rate-limiting enzyme for creatine synthesis, is up-regulated in GBM, which is reported to mediate de novo synthesis of creatine, and enhance cancer metastasis and shortens mouse survival [22].

In addition, our data revealed that OGDHL (oxoglutarate dehydrogenase like), a main rate-limiting subunit of the 2-oxoglutarate dehydrogenase multienzyme complex, was significantly down-regulated in GBM. OGDHL is involved in the tricarboxylic acid cycle, and frequently down-regulated in human carcinoma and suppresses tumor growth, which could be a predictive factor for worse prognosis in patients with pancreatic cancer [23].

## 4. Limitations

This study has some limitations. The quantitative proteomics experiment were performed based on six GBM patients. Even most of the proteomic changes were consistent with the RNA-seq datasets, future studies on a larger cohort may still be needed. In addition, considering the tumor heterogeneity and pathogenesis complexity of GBM, the mechanism of mitochondrial dysfunction still needs to be clarified by in vitro and/or animal studies in the future.

## 5. Conclusions

This study profiled the mitochondrial dysfunctions in GBM by revealing mitochondria-specific proteomic alterations in human GBM, and combining them with the transcriptional changes. The study revealed significant changes in GBM, including up-regulations of mitochondrial translation and apoptosis, as well as down-regulations of OXPHOS and mitochondrial dynamics. In addition, FA, AA metabolism and ROS also showed significant variations. Most of the alterations were consistent at protein and mRNA level, except for a few proteins/genes, and the changes at protein levels seems to be relatively more dramatic. This study would provide a useful resource to the community for further thorough analyses, including elucidating the pathological mechanism, or developing new therapeutic interventions or drugs for GBM.

## 6. Materials and Methods

### 6.1. Patients and Tissue Samples

Approval was obtained from the Ethics Committee of Tongji Hospital, Tongji Medical College, Huazhong University of Science and Technology in Wuhan. All the procedures involving human samples conformed to the principles outlined in the Declaration of Helsinki. Participation was voluntary and informed consent obtained in all cases. Tumor and peritumoral tissue (as paired control) samples of primary glioblastoma multiforme (GBM) were collected from the same six patients undergoing surgery at the Department of Neurosurgery of Tongji Hospital. All samples were de-identified. Samples were flash frozen and processed for proteomic analysis.

For the six patients, GBM was diagnosed by CT/MRI, followed by histopathology for confirmation. The mean age is 51 years (from 36 to 62 years), and two of the six were women, and two have co-morbidities of hypertension. All the GBM were IDH1 wild-type. The primary site was lateral ventricles (two cases), temporal lobe (two cases) and frontal lobe (two cases). The detailed characteristics of all the six patients were outlined in Table S1.

### 6.2. Mitochondrial Isolation

GBM and the paired peritumoral tissue (as control) from the same six patients were used for mitochondrial proteomics analysis. The mitochondria fractions were prepared using the Tissue Mitochondria Isolation Kit (Beyotime, Shanghai, China), according to the manufacturer’s instructions. Briefly, 50 mg of tumor or control tissue was weighed into a cold centrifuge tube and cut into very fine tissue fragments. The tissue was digested with trypsin digestion solution for 20 min followed by the addition of mitochondrial separation reagent, then homogenized on ice for ~20 times. The tissue was centrifuged for 5 min at 600× *g* and 4 °C, and then the supernatant was transferred and centrifuged for 10 min at 11,000× *g*, 4 °C. The mitochondrial pellets were collected and stored at −80 °C.

Purification of mitochondrial fractions was confirmed by detecting mitochondria marker proteins TOM20 and COX 4 (cytochrome oxidase IV), and nuclear protein lamin A/C, Histone H3, cytoplasmic protein β-actin and Hsp90 in the mitochondrial fractions, cytoplasm fraction and total lysate, respectively. As shown in Figure S1, TOM-20 and COX 4 were found to be obviously enriched in the mitochondrial fractions, while the Lamin A/C, Histone H3, β-actin, Hsp90 were almost not detected in the fractions.

### 6.3. Protein Digestion

Protein digestion was performed as previous reported with modifications [24,25]. Briefly, the mitochondrial fractions were lysed in lysis buffer consisting of 2.5% SDS/100 mM Tris-Cl (pH 8.0). Raw protein solution was obtained from lysate by sonicating for 10 min in ice-water bath, and centrifugation for 15 min at 12,000× *g* (4 °C). The protein in the supernatant was precipitated using acetone. The protein was then dissolved in the buffer consisting of 8 M Urea/100 mM Tris-HCl (pH 8.0). After centrifugation, the supernatant was used for reduction reaction (10 mM DTT, 37 °C for 1 h), and followed by alkylation reaction (40 mM iodoacetamide, room temperature/dark place for 30 min). Protein concentration was measured by Bradford method. Urea was diluted below 2 M using 100 mM Tris-HCl (pH 8.0). Trypsin was added at a ratio of 1:50 (enzyme: protein, *w*/*w*) for overnight digestion at 37 °C. The next day, TFA was used to bring the pH down to 6.0 to end the digestion. After centrifugation (12,000× *g*, 15 min), the supernatant was subjected to peptide purification using Sep-Pak C18 desalting column. The peptide eluate was vacuum dried and stored at −20 °C for MS detection.

### 6.4. LC-MS/MS (DIA) Detection

LC-MS/MS data acquisition was carried out on a Q Exactive HF mass spectrometer coupled with UltiMate 3000 RSLCnano system (both Thermo Scientific, Carlsbad, CA, USA) [26]. Peptides were first loaded onto a C18 trap column and then eluted into a self-made C18 analytical column (75 μm × 250 mm, 1.9 μm particle size, 100 Å pore size, Dr. Maisch). Mobile phase A (3% DMSO, 97% H_2_O, 0.1% formic acid) and mobile phase B (3% DMSO, 97% ACN, 0.1% formic acid) were used to establish a 60 min gradient. A constant flow rate was set at 300 nL/min. For data-independent acquisition (DIA) analysis, each scan cycle consisted of one full MS scan (R = 60 K, AGC = 3 × 10^6^, max IT = 30 ms, scan range = 350–1250 m/z) followed by 32 variable window DIA MS/MS scans (R = 15 K, AGC = 1 × 10^6^, max IT = 50 ms). HCD collision energy was set to 28.

### 6.5. Proteomic Data Processing

DIA-MS raw data were analyzed using DIA-NN (V1.8) software [27]. A spectral library for the library-free search was generated from the SwissProt human protein sequence database downloaded from Uniport (https://www.uniprot.org/, accessed on 11 October 2022). The raw data were processed using DIA-NN using the high accuracy quantification strategy with RT-dependent cross-run normalization enabled. The search parameters were as follows: protease, trypsin/P; missed cleavages, 1; static modification, cysteine carbamidomethylation; and enabled “Remove likely interferences” and “Use isotopologues”. Additional commands were set to -relaxed-prot-inf. The protein identification threshold was set at <1% false discovery rate (FDR).

For DIA data quantification, the output file generated by DIA-NN for each GBM or control tissue sample was processed in the R (version 4.2.1) workspace and used for the downstream analysis. Proteins with only one identified peptide were removed from the quantification matrix. Protein quantification was subsequently carried out using the MaxLFQ algorithm implemented in DIA-NN. The intensity values were log2-transformed for calculation and statistical analysis. For data filtration, a strict filtering rule was set as that, only proteins detected simultaneously in all GBM and peritumoral specimens were retained for quantification. Next, the quantification matrix was subjected to a global median normalization to normalize out the technical differences.

### 6.6. RNA-Seq Datasets

The RNA-seq data of GBM and normal brain tissue were downloaded from the “TCGA TARGET GTEx” cohort of the UCSC Xena (2016-09-03 version, TcgaTargetGtex_gene_expected_count) (https://xenabrowser.net/datapages/, accessed on 12 October 2022) [28]. It is a composite database derived from healthy people and cancer patients. This allows us to perform the analysis of the gene expressions on the 166 samples of GBM tumor from TCGA and 206 samples of normal brain tissues data (the cortex, frontal cortex) from GTEx. Transcript expression was quantified using RSEM. The RSEM expected counts, The Cancer Genome Atlas (TCGA) survival data, and phenotypic data were obtained. RSEM expected counts were provided as log2 (expected_count + 1) transformed, and in this study, this transformation was removed to produce raw expected counts for us. The expected count matrix was used to sort differently expressed genes by Limma R package.

### 6.7. Catalogue of Gene Symbols for Human Mitochondrion

For mitochondrial analysis, the MitoCarta3.0, a catalogue of gene symbols for human mitochondrion with sub-organelle localization and pathway annotations [29], was used to map the genes from our proteomics dataset and the RNA-seq dataset. MitoCarta3.0 provides an updated inventory of all protein components resident in the mammalian mitochondrion, which compiled using a Bayesian integration of multiple sequence features and experimental datasets, notably protein mass spectrometry of mitochondria.

### 6.8. Bioinformatics

In our DIA-MS based proteomics, to identify the differentially expressed proteins between GBM and its paired peritumoral control tissue, statistical significance was assessed by paired t-test. Proteins with *p* value < 0.05 and fold change (FC) between GBM/control of each protein >2.0 or <0.5 were considered to be significantly changed. In RNA-seq data analysis, to identify the differentially expressed genes, statistical significance was assessed by a simple linear model and moderated t-statistics using an empirical Bayes shrinkage method performed by the Limma R package [30]. Genes with adjusted *p* value < 0.05 and fold change (FC) >2 or <0.50 were considered to be significantly changed. All statistical analysis was performed using R (version 4.2.1).

Hierarchical clustering was applied to explore the differences in expression patterns between the GBM or control samples, and between the mitochondrial proteins/genes, using the R heatmap package. Functional enrichment analysis of differentially expressed mitochondrial proteins was based on Gene Ontology (GO) and Kyoto Encyclopedia of Genes and Genomes (KEGG) pathway database and using R package ClusterProfiler [31]. The protein–protein interaction (PPI) network was conducted via STRING database (https://string-db.org/, accessed on 11 October 2022). Cytoscape 3.7.2 (https://js.cytoscape.org/, accessed on 11 October 2022) was used to the visualize PPI networks. Survival analysis was performed by evaluating the prognostic significance of the DEGs in GBM using a Kaplan–Meier curve in Gene Expression Profiling Interactive Analysis (GEPIA) [32]. In the analysis, the median expression value of each DEG was used as a cut-off value to separate the samples into high/low expression groups. The Cox proportional hazards model was used to calculate the *p* value.

## Data Availability

The datasets used and/or analyzed during the current study available from the corresponding author on reasonable request.

## Supplementary Materials

The following supporting information can be downloaded at: https://www.mdpi.com/article/10.3390/molecules28041595/s1, Figure S1: Purification of mitochondrial fractions were confirmed by westrn blotting.; Table S1. Patient infomation in DIA-MS based proteomics.; Table S2. Summary of the relative intensities for the total quantified proteins.; Table S3. Summary of differentially expressend proteins (DEP); Table S4. Summary of differentially expressend mitochondrial proteins (DIA-MS) and mRNA (RNA-seq).

## Abbreviations

DIA-MS: Data-independent acquisition mass spectrometry; GBM, Glioblastoma multiforme; OXHPOS, oxidative phosphorylation; TCGA, The Cancer Genome Atlas; GEPIA, Gene Expression Profiling Interactive Analysis; FC, fold change; GO, Gene Ontology; KEGG, Kyoto Encyclopedia of Genes and Genomes; PCA, Principal Component Analysis; PPI, protein–protein interaction; DEG, differentially expressed genes; DEP, differentially expressed proteins; ROS, Reactive oxygen species; ALDH, aldehyde dehydrogenase; FA, fatty acid; AA, amino acid.
