# Supplementary material for: DIA-MS Based Proteomics Combined with RNA-Seq Data to Unveil the Mitochondrial Dysfunction in Human Glioblastoma
Source: Molecules. 2023 Feb 7;28(4):1595. doi: 10.3390/molecules28041595 (PMC9967398; doi:10.3390/molecules28041595)
Supplement: Supplementary file 1 [file molecules-28-01595-s001.zip › Supplementary_Figure_S1_(Fig.S1).pdf]

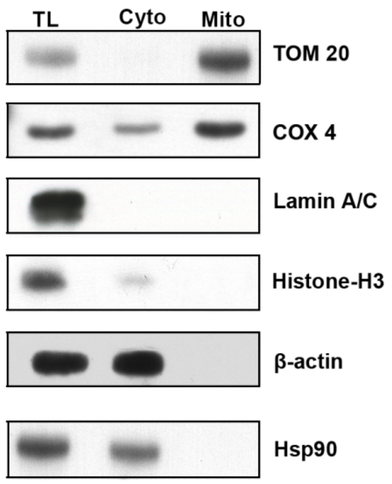

Figure S1. Purification of mitochondrial fractions were confirmed by detecting mitochondria marker proteins TOM20 and COX 4, and nuclear protein Lamin A/C, Histone H3, cytoplasm protein β-actin and Hsp90. TL: total lysate, Cyto: cytoplasm fraction, Mito: mitochondria.
